# Platelet function in patients undergoing major non‐cardiac vascular surgery (PLUGS): a prospective cohort study*

**DOI:** 10.1111/anae.70234

**Published:** 2026-05-13

**Authors:** Akshay Shah, Grace Polley, Kasia Bera, Keith Maher, Stephen Von‐Kier, Antonio Barbosa, Louis Corrigan, Sabeena Sharma, Peter Baker, Stuart R. McKechnie, Michael J. Desborough, the PLUGS Investigators

**Affiliations:** ^1^ Department of Anaesthesia Imperial College Healthcare NHS Trust London UK; ^2^ Nuffield Department of Clinical Neurosciences University of Oxford Oxford UK; ^3^ Oxford Critical Care Oxford University Hospitals (OUH) NHS Foundation Trust Oxford UK; ^4^ Department of Vascular Surgery OUH NHS Foundation Trust Oxford UK; ^5^ Nuffield Department of Surgical Sciences University of Oxford Oxford UK; ^6^ Haemostasis and Blood Conservation Service Oxford University Hospitals NHS Foundation Trust Oxford UK; ^7^ University of Oxford Medical School Oxford UK; ^8^ Nuffield Department of Anaesthetics OUH NHS Foundation Trust Oxford UK; ^9^ Oxford Haemophilia and Thrombosis Centre Nuffield Orthopaedic Centre Oxford UK; ^10^ Radcliffe Department of Medicine University of Oxford Oxford UK

**Keywords:** haemostasis, peri‐operative medicine, platelets, vascular

## Abstract

**Introduction:**

Patients who require major vascular surgery often receive antiplatelet therapy for primary or secondary prevention of cardiovascular disease. Clopidogrel resistance and variability in platelet recovery after drug discontinuation pose clinical challenges, particularly for regional anaesthesia and blood management. The aim of this study was to characterise platelet function and determine the prevalence of antiplatelet resistance using near‐patient viscoelastic testing in patients undergoing major, elective non‐cardiac vascular surgery.

**Methods:**

We conducted a single‐centre, prospective, observational cohort study at a tertiary vascular surgery centre. Adults scheduled for elective vascular surgery were recruited into four groups: aspirin; clopidogrel; dual antiplatelet therapy; and control (no antiplatelet therapy). Blood samples were obtained at pre‐operative assessment and on the day of surgery. Platelet function was assessed using thromboelastography and von Willebrand factor antigen levels. The primary outcome was the proportion of patients with antiplatelet resistance.

**Results:**

Eighty patients were enrolled, of whom 64 proceeded to surgery. Antiplatelet resistance was common, affecting 25–70% of patients at baseline depending on regimen and 15–83% on the day of surgery. Clopidogrel resistance was most frequent (70%). Two patients experienced early graft or stent thrombosis, both with evidence of clopidogrel resistance and elevated von Willebrand factor; von Willebrand factor levels exceeded the normal range in two‐thirds of patients. In an exploratory analysis, clopidogrel cessation 5–7 days pre‐operatively did not result in a statistically significant change in platelet inhibition.

**Discussion:**

High rates of clopidogrel resistance and elevated von Willebrand factor were observed in patients with planned vascular surgery, suggesting that current peri‐operative discontinuation guidelines may not restore normal platelet function. Larger multicentre studies that incorporate standardised platelet and near‐patient genetic testing are required to validate these findings and guide personalised peri‐operative antiplatelet management.

## Plain Language Summary

**What we did:**

We studied people who were having major blood vessel surgery. Many of them were taking medicines that slow blood from clotting, such as aspirin or clopidogrel, sometimes called "blood thinner" drugs. We took blood samples before surgery and on the day of surgery to see how well their platelets (the parts of blood that help it clot) were working.

**Why did we do it:**

These medicines are important for preventing heart problems, but they can also make surgery more difficult because of bleeding or clotting risks. Sometimes the medicines do not work properly in some people, or their effects do not wear off as expected. We wanted to understand how often this happens so doctors can plan safer care.

**What we found:**

We found that many patients did not respond to their blood‐thinner medicines as expected, especially those taking clopidogrel. In some people, the blood was still too likely to clot, even after stopping the medicine before surgery. We also found high levels of a blood protein linked to clotting in many patients. This suggests that current advice about when to stop these medicines may not always work, and better ways to check blood clotting before surgery may be needed.

## Introduction

Increasing numbers of patients who present for non‐cardiac vascular surgery are prescribed antiplatelet therapy for primary or secondary prevention of cardiovascular disease. The most common antiplatelet drugs in this cohort are aspirin and clopidogrel. Both are associated with increased peri‐operative bleeding [1] and increased peri‐procedural bleeding risk. Clopidogrel, in particular, may increase the risk of epidural haematoma when central neuraxial anaesthesia is performed [2, 3]. Epidural haematoma is a rare but serious complication of neuraxial anaesthesia, with an estimated incidence of 1.6 (95%CI 1.0–3.7) events per 100,000 cases [4].

Patients who require major vascular surgery are increasingly older; living with multimorbidity; and have higher ASA physical status scores [5]. In some patients, particularly those with underlying lung disease, the benefits of epidural anaesthesia/analgesia may outweigh the risk of an epidural haematoma [6, 7]. Current guidelines suggest continuation of aspirin monotherapy before neuraxial blockade but recommend discontinuation of clopidogrel (either as monotherapy or as part of dual antiplatelet therapy) 5–7 days before surgery. Current guideline recommendations do not consider individual variability in pharmacodynamic and pharmacokinetic response, but acknowledge that this is an important evidence gap [8, 9, 10]. Approximately one‐third of patients who take clopidogrel are classified as non‐responders because of impaired ability to metabolise the pro‐drug clopidogrel to its active metabolite. Platelet recovery profiles following discontinuation of clopidogrel also appear variable [11].

In view of these uncertainties, the leading question in a previous research priority setting exercise conducted by the Vascular Anaesthesia Society of Great Britain and Ireland was ‘*Can regional anaesthesia safely be performed on patients taking clopidogrel and similar antiplatelet agents?*’ However, it is challenging to define the magnitude of this risk in patients who take antiplatelet drugs in clinical practice. The gold standard method of assessing platelet function is light transmission aggregometry, but this is a time‐consuming, labour‐intensive method and requires a large volume of blood in comparison with other methods [11]. Thromboelastography 6 s (TEG®, Haemonetics Corp, Boston, MA, USA) has been approved for clinical use [12, 13, 14] and provides near‐patient, real‐time information on the viscoelastic properties of clot formation, together with information on platelet mapping and percentage inhibition [15, 16]. The primary objective of this exploratory study was to characterise platelet function in patients who required major, non‐cardiac, vascular surgery using a combination of near‐patient viscoelastic testing and TEG 6 s.

## Methods

This report was prepared according to STROBE guidelines. The ‘Platelet function in patients undergoing major non‐cardiac vascular surgery’ (PLUGS) study was a single‐centre, prospective, non‐interventional cohort study conducted at a tertiary vascular surgery centre (John Radcliffe Hospital, Oxford, UK). The study protocol was approved by the West Midlands – Solihull NHS Research Ethics Committee.

Potential patients were identified during pre‐operative assessment clinics, vascular triage and/or multidisciplinary team meetings. Inclusion criteria were: age > 18 y; scheduled to undergo elective major, non‐cardiac, vascular surgery (carotid endarterectomy, abdominal aortic aneurysm (open or endovascular), lower limb arterial revascularisation (femoral endarterectomy or bypass) or lower limb amputation); and able to provide written, informed consent. Patients should have been prescribed antiplatelet therapy – aspirin alone, clopidogrel alone or dual antiplatelet therapy – for at least 7 days before recruitment. Patients recruited to the control group did not receive any antiplatelet therapy. Key exclusion criteria were congenital or inherited bleeding disorders and haemodialysis‐dependent chronic kidney disease. Patients who were on anticoagulant therapy (e.g. warfarin, direct oral anticoagulants) were included but discussed on a case‐by‐case basis.

A schedule of study procedures is in online Supporting Information Figure S1. Study‐specific blood samples were collected at clinic and on the day of surgery. To minimise patient discomfort, samples were taken during aspects of routine care where possible, e.g. whilst obtaining routine blood tests or after venous cannulation before induction of anaesthesia. The TEG 6S assays were run in accordance with manufacturer guidelines by a trained member of the research team in the operating theatres within 20–40 min of sampling. Blood for von Willebrand factor (VWF) antigen (Ag) assessment was drawn into 0.109 M sodium citrate. Blood samples were centrifuged at 1760 g for 20 min and the plasma was aspirated and frozen at ‐80°C. The samples were analysed in batches using VWF:Ag reagents (vWF Ag® Kit, Siemens, Marburg, Germany) on a fully automated Sysmex CS‐5100 analyser (Siemens, Erlangen, Germany).

The primary outcome was the proportion of patients with antiplatelet drug resistance at initial presentation to clinic and on the morning of the surgery. For aspirin, drug resistance was defined as a mean amplitude arachidonic acid (MA_AA_)‐induced platelet‐fibrin clot strength > 47 mm plus an AA‐induced platelet inhibition rate < 50% (18). For P2Y_12_ inhibitors (clopidogrel), drug resistance was defined as a mean amplitude adenosine diphosphate (MA_ADP_)‐induced platelet‐fibrin clot strength > 47 mm plus an ADP‐induced platelet inhibition rate < 50% [17].

Secondary outcomes included: proportion of patients who self‐reported being compliant with antiplatelet medications pre‐operatively (compliance defined as taking > 80% of prescribed doses); peri‐operative blood loss and transfusion requirements within the first 24 h; return to the operating theatre and/or graft or stent thrombosis within the first 24 h; bleeding complications associated with regional anaesthesia; peri‐operative thrombotic events within 30 days of surgery (definitions provided in online Supporting Information Table S1); in‐hospital mortality; and readmissions within 30 days of hospital discharge. An exploratory outcome was the proportion of patients with low VWF Ag or VWF Ag levels in the normal reference range (0.5–1.0 iu.ml^‐1^).

Our centre performs more than 300 major vascular cases per year. In this exploratory study, we aimed to recruit 80 patients over 52 weeks (1–2 per week on average) and compare platelet inhibition across each of the following study groups: aspirin only; clopidogrel alone; dual antiplatelets; and no antiplatelets (acting as a control group). As an exploratory observational study, no formal sample size calculation was undertaken. Based on previous work [18], we planned to include 20 patients in each group on the basis that the information obtained from this study would be used to inform the sample size calculations for a future, multicentre study.

Continuous variables were assessed for normality with visual inspection of histograms and using the Shapiro–Wilk test. Fisher's exact test, the independent samples t‐test or the Mann–Whitney U test were used, as appropriate, to determine differences in percentage platelet inhibition across the study groups. Repeated measures ANOVA was used to compare platelet inhibition in patients who required sampling to two time‐points. Analyses were conducted on the intention‐to‐treat and per‐protocol populations. Tests were two‐sided and considered to provide evidence for a statistically significant difference if p values were < 0.05. All analyses were undertaken using Stata (version 15.1, STATACorp LP, College Station, TX, USA).

## Results

**Figure 1:**
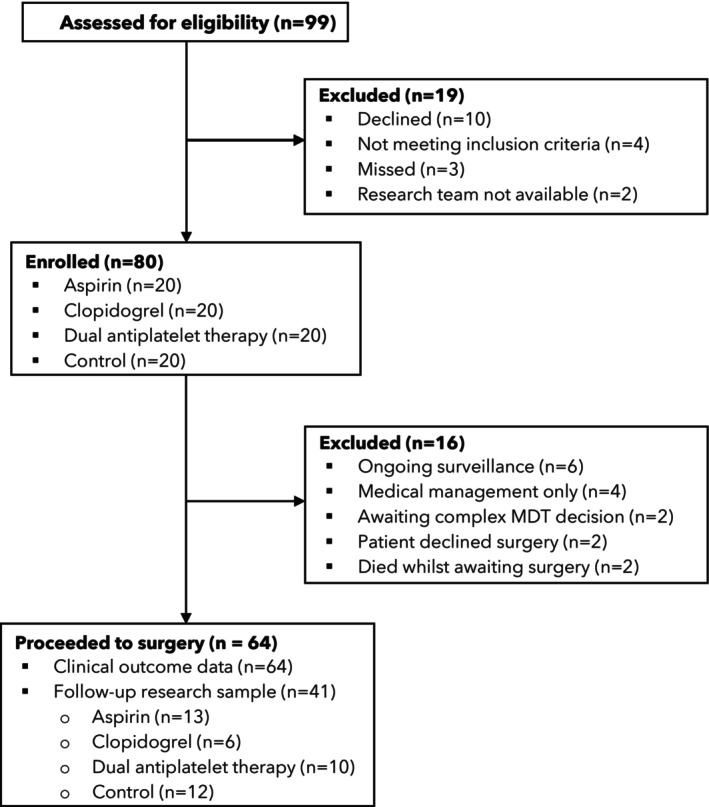
Flow diagram of patient enrolment.

Between 9 June 2022 and 17 April 2023, 80 patients were enrolled (Fig. 1). Of these, 64 (80%) continued to have their planned surgery. Baseline characteristics, surgical procedures and pre‐operative laboratory values for the full study cohort and according to study group are in Table 1 and online Supporting Information Table S2. Details of changes to antiplatelet medication are shown in online Supporting Information Table S3. Adequate compliance with medications in the preceding 2 weeks was reported by 56/60 (93%) of patients (online Supporting Information Table S4). Details of mode of anaesthesia are in online Supporting Information Table S5.

**Table 1:** Baseline characteristics of patients. Values are mean (SD).

	Aspirin n = 20	Clopidogrel n = 20	Dual antiplatelet therapy n = 20	Control n = 20
Age; y	71 (7.8)	74 (12.1)	75 (6.4)	67 (17.8)
Sex; male	17	16	16	20
Weight; kg	82 (14.0)	81 (17.9)	83 (12.0)	82 (20.0)
Ethnicity				
White	20	19	20	15
Mixed				1
Asian		1		2
Black				1
Other				1
ASA physical status				
2	3	2	5	4
3	16	17	15	16
4	1	1	–	–
Comorbidities				
1	4	–	2	2
2	4	2	8	6
3	3	7	6	3
4	6	6	1	3
≥ 5	3	5	3	6
Blood tests				
Haemoglobin; g.l^‐1^	138 (17.1)	131 (36.2)	124 (41.5)	142 (15.5)
Platelet count; ×10^9^.l^‐1^	289 (124)	268 (108)	368 (140)	281 (133)
Creatinine; μmol.l^‐1^	92 (29.3)	81 (27.0)	92 (28.1)	80 (21.1)
Prothrombin time; s	10.6 (0.5)	11.3 (2.9)	10.5 (0.48)	11.0 (0.89)

Table 2 summarises TEG 6 s values of patients on antiplatelet therapy and those of the control group from samples taken in clinic. According to our prespecified definitions, the proportion of patients with antiplatelet resistance ranged from 25% to 70%, depending on antiplatelet regime. Antiplatelet resistance was observed in 14/20 patients on clopidogrel. Of samples available from the day of surgery, the proportion of patients with antiplatelet resistance ranged from 15% to 83% (Table 3). Approximately two‐thirds of all patients (53/80, 66%) had VWF levels above the normal range (> 1.50 iu.ml^‐1^).

**Table 2:** Platelet function at baseline/pre‐operative clinic visit. Values are mean (SD) or number.

	Aspirin n = 20	Clopidogrel n = 20	Dual antiplatelet therapy n = 20	Control n = 20
MA_AA_; mm	39.7 (15.2)	43.8 (17.9)	28.1 (16.8)	61.1 (5.1)
AA‐inhibition; %	48.9 (28.5)	38.4 (35.7)	68.0 (31.9)	9.8 (16.7)
MA_ADP_	58.9 (11.3)	54.8 (11.1)	47.8 (16.5)	55.8 (11.4)
ADP‐inhibition; %	9.9 (13.1)	18.1 (16.9)	31.5 (31.0)	10.1 (19.1)
von Willebrand factor	1.7 (0.51)	2.0 (1.2)	1.89 (0.5)	1.6 (0.5)
Patients with antiplatelet resistance	6	14	5	NA

MA, maximum amplitude; AA, arachidonic acid; ADP, adenosine diphosphate; NA, not applicable.

**Table 3:** Platelet function on day of surgery. Values are mean (SD) or number.

	Aspirin n = 13	Clopidogrel n = 6	Dual antiplatelet therapy n = 10	Control n = 12
MA_AA,_; mm	44.6 (13.9)	37.5 (22.1)	27.5 (17.0)	61.8 (3.3)
AA‐inhibition; %	36.5 (25.3)	64.0 (48.6)	70.6 (35.8)	11.9 (24.7)
MA_ADP_	61.6 (5.4)	51.3 (11.6)	49.9 (11.1)	47.2 (21.2)
ADP‐inhibition; %	6.4 (7.9)	31.0 (14.4)	27.9 (22.2)	14.8 (23.3)
Patients with antiplatelet resistance	2	5	3	NA

MA, maximum amplitude; AA, arachidonic acid; ADP, adenosine diphosphate; NA, not applicable

Two patients (2/64; 3%) experienced graft or stent thrombosis resulting in acute limb ischaemia in the first 24 h. Both had evidence of pre‐operative clopidogrel resistance and elevated VWF levels. No patients experienced peri‐operative thrombotic complications (not related to stent or graft thrombosis) within 30 days of surgery as per our prespecified definitions (online Supporting Information Table S6). Of the 13 patients who received regional anaesthesia, one required minor compression for bleeding. Across the whole cohort, two patients died whilst in hospital and seven patients were readmitted.

**Figure 2:**
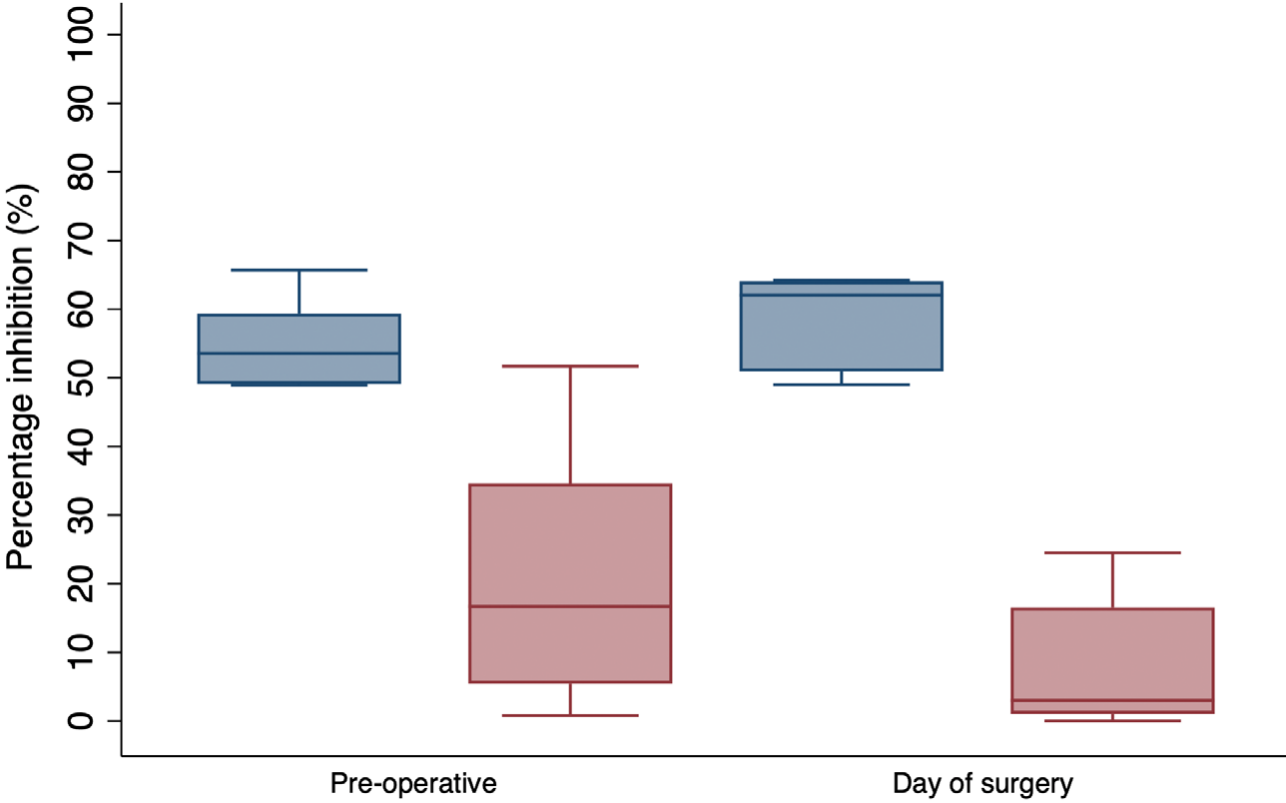
Changes in platelet resistance in patients who were advised to stop clopidogrel before surgery (n = 8). Boxplots show maximum amplitude adenosine diphosphate (MA_ADP_) and ADP‐inhibition measured pre‐operatively and on the day of surgery. Boxes indicate interquartile range, central line median and whiskers minimum to maximum values. Blue, MA_ADP_ values; red, adenosine diphosphate inhibition.

In a post‐hoc exploratory analysis, we compared pre‐operative assessment clinic and day of surgery samples in eight patients who were advised to stop clopidogrel 5–7 days before surgery. We found no evidence of an effect of stopping clopidogrel on MA_ADP_ (mean (SD) 56.1 (11.7) vs. 52.7 (10.7) mm, mean difference (MD) 95%CI 3.4 (16.3 to ‐10.2), p = 0.57) or ADP‐inhibition (10.6 (15.5) vs. 20.7 (18.6) %, MD 95%CI ‐10.3 (18.3 to ‐25.4), p = 0.16) (Fig. 2 and online Supporting Information Table S7).

## Discussion

This exploratory study confirms a high prevalence of antiplatelet resistance, particularly to clopidogrel, in patients who present for vascular surgery in a real‐world setting. In addition, we also found that VWF levels were elevated frequently and that discontinuation of clopidogrel 5–7 days before surgery did not appear to restore platelet responsiveness in an exploratory analysis.

This is important because peripheral vascular disease affects more than 200 million people worldwide [19] with antiplatelet and/or antithrombotic therapy a key component of medical therapy. Current guideline‐based recommendations do not consider individual variability in response to treatment. For patients with symptomatic lower extremity arterial disease, current European Society for Vascular Surgery guidelines recommend clopidogrel (75 mg) as first line therapy (class 1 recommendation) or aspirin (75 mg) and low‐dose rivaroxaban (2.5 mg twice a day) for patients at high ischaemic risk [20]. However, these guidelines acknowledge the paucity of data on patients with demonstrable evidence of platelet resistance (or high‐on‐treatment platelet reactivity) and its association with clinical outcomes.

Our findings align with previously published work. Systematic reviews and observational studies show that the prevalence of clopidogrel resistance ranges from 0% to 33% depending on the assays and cut‐off values used [21, 22]. The higher rates reported in our study may, in part, be explained by population‐specific risk factors (e.g. multimorbidity, chronic inflammation). Antiplatelet resistance to clopidogrel, aspirin or both is associated with stent or graft thrombosis, amputation and need for re‐intervention in patients with peripheral vascular disease [23, 24]. In our study, both patients who experienced graft thrombosis had pre‐operative evidence of clopidogrel resistance.

In our unpowered post‐hoc exploratory analysis, we found that stopping clopidogrel according to guideline recommendations did not impact on markers of platelet resistance. Most patients in this analysis showed minimal baseline clopidogrel effect such that cessation would not be expected to result in substantial changes in platelet function. These findings therefore likely reflect underlying variability in clopidogrel responsiveness rather than true recovery of platelet function following drug discontinuation and should be considered hypothesis‐generating. At the same time, patients undergoing vascular surgery represent a population at particularly high cardiovascular risk, for whom effective long‐term antiplatelet therapy is essential for secondary prevention. Pre‐operative assessment clinics may therefore represent an important opportunity within the healthcare pathway to identify patients with suboptimal platelet inhibition or suspected drug resistance, and alternative antiplatelet strategies for longer term care should be considered.

An important limitation of the currently available evidence is the heterogeneity related to different surgical procedures, diversity in the drugs used with respect to dose and duration and inconsistent outcome reporting. Several devices for platelet function testing are now available but there is lack of consistency and standardisation across different assays. TEG platelet mapping provides a reproducible bedside assessment of platelet function. Several devices for platelet function testing are now available, but there remains a lack of consistency and standardisation across assays. Light transmission aggregometry has been regarded historically as the gold standard. Near‐patient techniques such as TEG platelet mapping offer practical advantages and reproducible bedside assessment but may quantify platelet inhibition differently. This methodological variability complicates comparison across studies and underscores the need for harmonised testing approaches and validation against established reference methods.

The contribution of VWF towards primary haemostasis is not measured by most standard platelet function tests. High levels of VWF may contribute towards antiplatelet drug resistance [25]. Patients with atherosclerotic vascular disease also show a chronic low‐level systematic inflammatory phenotype characterised by elevated C‐reactive protein, interleukin‐6 and VWF levels [26, 27]. Similarly, it is recognised that an ABO blood group influences VWF levels and platelet–VWF interactions, with reduced binding observed in patients with blood group O, potentially contributing to variability in platelet aggregation and thrombotic risk [28, 29]. Platelet aggregation is also heritable and may vary across ancestral groups, highlighting the need for larger, diverse cohorts to explore the influence of ABO genotype and ancestry on antiplatelet response [30]. Population‐level data suggest that three in five adults with vascular disease have systemic inflammation, which is associated with increased healthcare use and rates of major adverse cardiovascular events [27].

Variability in antiplatelet response is not only relevant in the peri‐operative setting but also in broader cardiovascular practice, where high‐on‐treatment platelet reactivity has been associated with increased risk of myocardial infarction, stroke and stent thrombosis [31]. Consensus guidelines suggest that platelet function testing, including genotype testing, could inform personalised antiplatelet therapy in high‐risk patients [32]. This wider clinical impact further emphasises the importance of understanding differential antiplatelet responsiveness.

For patients who experience bleeding whilst on antiplatelet drugs, augmentation of VWF with desmopressin has been proposed as a strategy to improve haemostasis. Desmopressin reduces bleeding in cardiac surgery [33] and is under investigation for reversing the antiplatelet drug effect in intracerebral haemorrhage [34]. However, its haemostatic effect may be attenuated when baseline VWF levels are already high [35] and therefore, in this context, the efficacy of desmopressin is likely to be reduced.

Our study has strengths and limitations. A key strength is the prospective design with high rates of follow‐up and contemporaneous collection of laboratory and near‐patient platelet function data to facilitate integration of clinical and mechanistic insights. However, as a single‐centre study the findings may not be generalisable. Our sample size was not powered to detect clinical outcome differences, but the estimates provided here will inform future sample size calculations. We did not perform platelet aggregometry for practical reasons, but future studies should incorporate both approaches to validate findings and establish their relevance to clinical outcomes. We did not collect data on medications that may contribute to iatrogenic drug resistance (e.g. omeprazole). We only measured VWF antigen alone rather than a full VWF activity profile; however, previous work in similar patient populations has shown close concordance between antigen, activity and collagen binding assays. Another limitation of our study is a lack of genetic data on CYP2C19 as this is associated with clopidogrel resistance [9]. An advantage of near‐patient tests such as TEG platelet mapping over genetic testing as a test of platelet function is that it benchmarks real‐time ex‐vivo platelet function and so is likely to determine the impact of other factors that may impact platelet function such as non‐compliance, uraemia or the presence of sepsis. The two technologies are likely to complement one another in clinical practice.

We recommend that future studies should extend our exploratory findings in larger, multicentre cohorts of patients who require vascular surgery using standardised platelet function assays and near‐patient genotype testing. This approach could better define the mechanisms and clinical relevance of antiplatelet resistance in vascular surgery. Recommendations for quality assurance for platelet function tests are available [36] and it is also important that local normal ranges are determined. Integrating real‐time platelet and genetic profiling with peri‐operative outcome data has the potential to guide personalised antiplatelet management strategies, including genotype‐guided prescribing [37]. This is particularly relevant as CYP2C19 loss‐of‐function alleles, which impair clopidogrel metabolism, are relatively common and have been associated with adverse cardiovascular outcomes in vascular surgery populations [38]. Further work should also explore the roles of systemic inflammation and elevated VWF levels in modulating platelet responsiveness and explore whether targeted approaches – such as genotype‐guided prescribing or modification of VWF activity – can improve peri‐operative bleeding and thrombotic outcomes.

In conclusion, this study highlights a high prevalence of antiplatelet resistance, particularly towards clopidogrel, among patients undergoing major vascular surgery and potentially suggests that discontinuation in line with current guidelines may not restore platelet responsiveness reliably. Larger multicentre studies, with near‐patient genetic testing, are now needed to confirm these findings and to inform peri‐operative antiplatelet management strategies.

## Supporting information


**Appendix S1.** PLUGS investigators.

**Figure S1.** Study flow chart.

**Table S1.** Definitions of peri‐operative thrombotic complications.
**Table S2**. Surgical procedures performed in each study group.
**Table S3**. Changes recommended at pre‐operative assessment clinic.
**Table S4**. Proportion of patients who self‐reported as being compliant with antiplatelet medications pre‐operatively.
**Table S5**. Mode of anaesthesia.
**Table S6**. Clinical outcomes.
**Table S7**. Change in platelet function in patients who were advised to stop clopidogrel 5–7 days pre‐operatively.
