# Supplementary material for: Platelet function in patients undergoing major non‐cardiac vascular surgery (PLUGS): a prospective cohort study*
Source: Anaesthesia. 2026 May 13;81(9):1236–44. doi: 10.1111/anae.70234 (PMC13446860; doi:10.1111/anae.70234)
Supplement: Supplementary file 1 [file ANAE-81-1236-s001.docx]

**Table S1.** Definitions of thromboembolic events.

| **Condition** | **Definition** |
| --- | --- |
| **Myocardial infarction** | Detection of an elevated cardiac troponin with at least one value above the upper reference limit in the context of myocardial ischemia and at least one of the following:  • Symptoms of myocardial ischaemia;  • New ECG changes indicative of ischaemia (for example ST segments changes or new left bundle branch block) or development of new T wave inversion or pathological Q waves  • Imaging evidence of new loss of viable myocardium or new regional wall motion abnormality |
| **Ischaemic stroke** | Evidence of a new infarction on a CT scan of the head and new neurologic deficit lasting at least 24 hours |
| **Venous thromboembolism** | Deep venous thrombosis confirmed by compression ultrasound, CT venogram or MR venogram; Pulmonary embolism confirmed by high probability ventilation perfusion scan or CT pulmonary angiogram' splanchnic vein thrombosis confirmed by CT abdomen or MRI of abdomen; or cerebral venous sinus thrombosis confirmed by CT venogram. |

**Table S2.** Surgical procedures performed in each study group.

|  | **All**  **(n = 80)** | **Aspirin**  **(n = 20)** | **Clopidogrel**  **(n = 20)** | **DAPT**  **(n = 20)** | **Control**  **(n = 20)** |
| --- | --- | --- | --- | --- | --- |
| Completed operation (n=64)  Carotid endarterectomy  Open AAA repair (infra-renal)  Open AAA repair (juxta-renal)  EVAR  Complex EVAR/FEVAR  Axillo-uni/bifemoral bypass  Aortobifemoral bypass  Femoral distal-bypass  Femoral popliteal bypass  Femoral endarterectomy  AKA | 20  10  2  8  3  1  3  2  2  6  7 | 0  5  0  2  1  0  3  0  1  1  2 | 7  1  1  0  1  1  0  1  1  1  2 | 11  1  0  1  0  0  0  1  0  1  1 | 2  3  1  5  1  0  0  0  0  3  2 |

AAA, Abdominal aortic aneurysm; AKA, Above knee amputation; DAPT, Dual antiplatelet therapy; EVAR, Endovascular aneurysm repair; FEVAR, Fenestrated endovascular aneurysm repair;

**Table S3.** Changes recommended at pre-operative assessment clinic.

| **Recommendation** | **n (%)** |
| --- | --- |
| Continue Aspirin until day of surgery | 19 (23.8) |
| Continue DAPT until day of surgery | 20 (25.0) |
| Stop Clopidogrel 5-7 days before surgery | 15 (18.8) |
| Switched from Clopidogrel to Aspirin | 5 (6.3) |
| Started on Aspirin and/or Clopidogrel | 1 (1.3) |
| Control group (i.e. no changes) | 20 (25.0) |

DAPT, Dual antiplatelet therapy

**Table S4.** Proportion of patients who self-report as being compliant with antiplatelet medications preoperatively. Compliance was defined as taking >80% of prescribed doses as prescribed.

| **Compliance** | n / total n (%) |
| --- | --- |
| Forget to take their antiplatelet therapy | 4/56 (7.1%, 95%CI 2.4-16.6) |
| Take their antiplatelet medication at the specified time | 14/60 (23.3%, 95%CI 13.8-35.5) |
| Stop taking the medication if they feel ill | 4/56 (7.1%, 95%CI 2.4-16.6) |
| Forget to take the medication during the weekend | 3/57 (5%, 95%CI 1.5-14.8) |
| No. of days forgotten to take medication in last 2 weeks  0  1-2  >2 | 52/56 (92.9%, 95%CI 83.3-98.5)  4/56 (7.1%, 95%CI 2.4-16.6) |

**Table S5.** Mode of anaesthesia

|  | n, number of patients |
| --- | --- |
| Mode of anaesthesia  General anaesthesia with volatiles  General anaesthesia with TIVA  Neuraxial anaesthesia  Regional anaesthesia  Sedation  Local infiltration  Unknown | 4  23  22  13  31  5  1 |

**Table S6.** Clinical outcomes

| **Outcome** | **Value** |
| --- | --- |
| Cross-clamp time (where applicable), min (median, IQR) | 43 (26-77) |
| Estimated blood loss, mls (mean (SD)) | 500 (100-1815) |
| Peri-operative transfusion   - RBC, n | 2 |
| Postoperative haemoglobin, g.l^-1^ (mean (SD)) | 114 (27.1) |
| Return to theatre, n | 1 |
| Graft/stent thrombosis in first 24 hours, n | 2 |
| Perioperative thrombotic complications up to 30 days (MI, CVA, DVT) | 0 |
| Acute limb ischaemia within 30 days, n | 0 |
| Complications with regional anaesthesia   - Bleeding requiring intervention (compression/interventional radiology) | 1 |
| Survived to hospital discharge, n (%) | 62 (96.8) |
| Readmitted within 30-days, n (%) | 7 (11.2) |

CVA, Cerebrovascular accident; DVT, Deep vein thrombosis; IQR, Interquartile range; MI, Myocardial infarction; n, number of participants; RBC, Red blood cell; SD, Standard deviation.

**Table S7.** Change in platelet function in patients who were advised to stop clopidogrel 5-7 days pre-operatively (n = 8).

|  | Pre-operative | Day of surgery | Mean difference (95%CI) | P value |
| --- | --- | --- | --- | --- |
| MA_ADP_  ADP-inhibition, % | 52.7 (10.7)  20.7 (18.6) | 56.1 (11.7)  10.6 (15.5) | 3.4 (16.3 to -10.2)  -10 (18.3 to -25.4) | 0.57  0.16 |
