# Supplementary material for: Platelet function in patients undergoing major non‐cardiac vascular surgery (PLUGS): a prospective cohort study*
Source: Anaesthesia. 2026 May 13;81(9):1236–44. doi: 10.1111/anae.70234 (PMC13446860; doi:10.1111/anae.70234)
Supplement: Supplementary file 2 [file ANAE-81-1236-s002.docx]

**Appendix S1: PLUGS Investigators**

Oxford University Hospitals NHS Foundation Trust

Neil Davidson

Jean Wilson

Soyamol Matthew

Sally Beer

Amanda Davies

Christopher Larkin

Sabina Wieczorek

Jenny Busian

Ping Zhang

Bea Vidotto

Pam Davis
